# The Novel Functions of M_4_ Muscarinic Receptors

**DOI:** 10.33549/physiolres.935750

**Published:** 2025-12-01

**Authors:** Jaromir MYSLIVECEK, Katerina JANISOVA

**Affiliations:** 1 Institute of Physiology, First Faculty of Medicine, Charles University, Prague, Czech Republic

**Keywords:** M_4_ muscarinic receptors, M_1_ muscarinic receptors, Biological rhythm, Locomotor activity, Striatum

## Abstract

The role of M_4_ muscarinic receptors in locomotor activity regulation remains controversial, with conflicting findings hampering our understanding of movement disorders. This uncertainty is further complicated by the unexplored relationship between M_4_ receptors and biological rhythms, as well as potential sex-specific effects that may explain previous inconsistent results. Through systematic investigation of locomotor functions in biological rhythm paradigms, we discovered that M_4_ muscarinic receptors significantly modulate locomotor activity rhythms *via* brain pacemaker mechanisms. Notably, this regulatory effect displayed marked sexual dimorphism, being present exclusively in females, suggesting crucial interactions with sex hormones. Our research identified the striatum as the key structure directing these locomotor biological rhythms. These findings provide critical insights into the sex-specific nature of M_4_ receptor function and establish a new framework for understanding hyperactivity disorders, particularly those exhibiting sex-based differences in prevalence or manifestation.

## Introduction

Muscarinic receptors are acetylcholine receptors that couple to G proteins. There are five subtypes of mAcChRs, designated M_1_ to M_5_. The activation or inhibition of mAcChRs plays a multifaceted role in the central nervous system, affecting motor coordination, cognitive function, attention, addiction, synaptic plasticity processes, and biological rhythms. In pathophysiology, mAcChRs are of importance in Alzheimer’s disease, Parkinson’s disease, bipolar disorder, schizophrenia, or depression (see [1]). The aim of this review is to demonstrate the novel M_4_ mAcChR functions in locomotor regulation and to show new aspect – the changes of the locomotion as function of the biological rhythm. Then we would like to shed the light on the structures responsible for the cyclic changes in the locomotion affected by M_4_ mAcChRs.

## Central cholinergic system

The central cholinergic system is a mediator system that includes neurotransmitters, the specific nuclei in which these substances are synthesized, and the pathways along which the impulse is conducted. Acetylcholine is found in both the central and peripheral nervous systems. It is synthesized from choline and acetyl coenzyme A with the help of choline acetyltransferase [2]. Acetylcholine is then transported by the vesicular transporter to the vesicles, where it is stored until it is needed for release into the synaptic cleft [3].

Acetylcholine released from cholinergic synapses is hydrolyzed by acetylcholinesterase (AChE) to choline and acetate, and nearly 50 % of the choline produced by hydrolysis of acetylcholine (ACh) is taken up by the high-affinity choline transporter [2]. Surprisingly, AChE is localized on the presynaptic membrane [4–6] and thus its function is rather regulatory than termination of ACh effects.

## Muscarinic receptors

Muscarinic acetylcholine receptors (mAcChRs) belong to a family of G protein-coupled receptors and regulate a diverse range of central and peripheral functions of the human body, including parasympathetic functions [7]. Centrally, they are involved in the regulation of cognitive, behavioral, motor, and autonomic functions. Peripherally, they mediate the action of acetylcholine in organs and tissues innervated predominantly by postganglionic parasympathetic nerves and participate in autonomic processes such as the regulation of heart rate, smooth muscle contraction, and exocrine and endocrine gland secretion. They then mediate non-neuronal actions of acetylcholine, including cell proliferation, differentiation, apoptosis, cell locomotion and migration, angiogenesis, immune function, secretion, cytoskeleton organization, and ciliary muscle activity [8]. The mAcChR family includes five subtypes – designated M_1_ to M_5_ and encoded by the CHRM1 to CHRM5 genes [9].

Different subtypes predominate in different tissues and organs [7]. They differ in distribution, preferential signaling pathways, and mediated physiological functions [10].

Muscarinic ligands can bind to two sites: orthosteric and allosteric [11]. Ligands able to bind to the orthosteric binding site of the receptor and thereby trigger its activation are called orthosteric agonists. Agonists bind to the orthosteric, essentially ‘main’ or active binding site with high affinity. The allosteric site then differs from the binding site for the endogenous ligand; it is a different binding site, physically distinct, and binding to this site then results in an enhancement or attenuation of the effect of the endogenous ligand. Normally, the binding of an allosteric modulator causes a conformational change in the receptor molecule, resulting in a change in the binding affinity of the ligand to the receptor. In this way, the allosteric ligand modulates the activation of the receptor by its primary (orthosteric) ligand [12].

The orthosteric binding site of all muscarinic receptor subtypes is virtually the same [13]. Finding agonists that differ in binding affinity for each subtype is very difficult. Moreover, orthosteric ligands can also bind to another target in the CNS [1]. The issue of the nature of the orthosteric binding site is addressed in the review by Randakova and Jakubik [14]. Agonist-specific conformations can lead to non-uniform modulation of signaling pathways. This preferential orientation of signaling toward one or a subset of its signal transducers is called agonist bias [11].

It is therefore evident that classical, orthosteric ligands (both agonists and antagonists) are not able to discriminate between muscarinic receptor subtypes. Thus, the search for muscarinic functions is limited to the studies using knockout animals or, as new, really selective ligands were discovered (see [1]), using newly synthesized, highly specific ligands [15–22].

## M_4_ muscarinic receptors in the CNS

M_4_ mAcChRs have been associated with various functions of the body over the past years. Initially, the roles of M_4_ mAcChRs were elucidated only in pharmacological studies, and thus important roles in brain processes such as learning and memory [23], attention [24], locomotion [25], thermoregulation, sleep, and wakefulness [26], food intake [27] and reward [28]. The selectivity of muscarinic receptor ligands commonly used at the time was limited, and the muscarinic receptor subtypes present in the tissue studied made it difficult to pinpoint a specific role. New light on the M_4_ mAcChRs function was shed on genetically modified mice deficient in M_4_ muscarinic receptors [29]. Subsequent studies performed on these biomodels were then able to reveal the consequences of M_4_ muscarinic receptor deletion in brain processes and helped to elucidate the molecular mechanisms that control neuronal circuits. Thus, M_4_ mAcChRs have been found to play an important role in behavior [30], social behavior [31], learning, memory, as well as addictions [32].

The control of movement involves neuronal circuits that are influenced by several receptors (muscarinic/nicotinic receptors, dopamine receptors, GABA receptors, and receptors for excitatory amino acids). The balance between them is an important factor for effective regulation of locomotion [29,33,34].

M_4_ muscarinic receptors can modulate neuronal circuits directly (postsynaptically) or indirectly by modifying acetylcholine tone by several mechanisms. Namely, by regulating neurotransmitter release, neuronal excitability, transcription, and translation [35]. Modulation of neurotransmitter release has been linked to the ability of M_4_ mAcChRs to cause long-term changes in synaptic strength [36]. Synaptic plasticity is thought to be a fundamental molecular mechanism of learning and memory [37].

The highest CNS number of M_4_ mAcChRs is found in the striatum. There is also a significant amount of M_1_ mAcChRs [38]. The cholinergic neurons also bear dopamine D_1_ and D_5_ receptors [39,40], and vice versa, dopaminergic neurons (with D_2_ dopamine receptors) bear M_3_ and M_5_ mAcChRs [40]. Another important number of M_4_ mAcChRs, together with M_1_ mAcChRs, is in the hippocampus [41]. Fewer M_4_ mAcChRs can be found in the cortex [42] or thalamus. Yasuda *et al*. detected the highest levels of M_4_ mAcChRs in the striatum and olfactory tubercle [43]. Dopamine receptors also belong to the GPCR family. M_4_ mAcChRs and D_1_ dopamine receptors have opposite regulatory effects, with M_4_ mAcChRs acting inhibitory to D_1_ dopamine receptors [29,39]. When M_4_ mAcChRs receptors are knocked out, D_1_ dopamine receptors are not inhibited, and locomotion is increased by activation of striatonigral pathways [39].

## M_4_ muscarinic receptors and locomotor activity

It is sometimes difficult to compare the types of motor activity that are followed in different studies (open-field locomotor activity in boxes or on plus mazes, circadian activity on running wheels, or in cages). In general, all these motor activities are directed by similar mechanisms, and thus, they could give us a picture of differences in motor activity between different groups of mice.

M_4_ mAcChRs have been shown to influence locomotor activity. The first study [29] has shown that M_4_ mAcChRs knockout significantly increases the overall animal motor activity. Initially, knockout studies were considered as an optimal method for the detection of gene function [44]. Nevertheless, the flanking allele effect was not always considered an important factor for behavior determination [45]. The subsequent review [46] described an increase in the overall animal motor activity, a slight effect on salivation, and no effect on tremor and hypothermia. The increased locomotion of M_4_ KO mice has been ascribed to the enhanced dopaminergic signaling at D_1_ dopamine receptors. Of note, initial studies were performed on mice with a mixed 129SvEv/CF-1 background. Further M_4_ KO study in which backcrossing was carefully performed on C57Bl/6J background, showed no basal M_4_ effects on motor activity [47]. This study was mainly focused on the anti-psychotic effects of xanomeline (muscarinic agonist) on amphetamine (able to increase noradrenaline, serotonin, and dopamine levels)-induced hyperactivity. Although the basal activity was not changed in M_4_ KO animals, amphetamine-induced hyperactivity was not attenuated in M_4_ KO animals. The next study then showed no change in the diurnal pattern of motor activity in M_4_ KO animals followed in a 24-hour cycle [48]. These mice were prepared on C57Bl/6NTac. Although the data were followed telemetrically, no biological rhythm analysis was made. The study with careful backcrossing, in animals made on a pure 129SvEv background, showed an increase in motor activity [31]. In detail, M_4_ KO mice exhibited novelty-induced locomotor hyperactivity in the open field test.

In the context of locomotor activity, it is necessary to stress that mice are nocturnal animals [49] and thus experiments performed in their non-active phase can be affected by this fact.

## Biological rhythms

Biological rhythms are cyclically repeating events in living organisms. These include, for example, sleep and wakefulness, body temperature, hormone secretion, metabolism, heart rate, blood pressure, urine production, and others [50]. These bodily functions are regulated by the body’s internal clock, the so-called central oscillator with electrical activity and a transcriptional-translational feedback loop [51]. The system oscillates spontaneously for a period of about 25 h. This process drives gene expression cycles in all major tissues, and these cycles are the basis of daily metabolic programs. These myriad cellular clocks are coordinated by a central pacemaker, the suprachiasmatic nucleus [52]. Many rhythmic parameters reflect the function of the central pacemaker – i.e., the suprachiasmatic nucleus (SCN). However, these parameters may also be influenced by the properties of other “clock” tissues [53].

The central oscillator is largely influenced by the alternation of light and dark; therefore, the basic rhythm is circadian, i.e., approximately 24 h. In humans, the circadian period varies depending on the environment, i.e., on synchronizing stimuli (activity, light in the room, or knowledge of the time of day) from 24.2 to 25.1 h. In the absence of periodic synchronizing stimuli from the environment, endogenous circadian rhythmicity persists with an internal period close to the rotation of the Earth [54]. Biological rhythms with a period longer than one day or 28 h are referred to as infradian, and rhythms with a period of less than one day or 20 h are referred to as ultradian.

Biological rhythms are maintained by synchronizing stimuli or, on the contrary, can be disturbed by them (e.g., light in dark periods, physical activity at inappropriate times, etc.). The suprachiasmatic nucleus of the hypothalamus (SCN) plays a major role in the daily timing of the body’s functions by regulating the daily oscillations of the internal environment and synchronizing them with the changing cycles of day and night and the state of the body [55]. The SCN is a hypothalamic paired structure on either side of the third cerebral ventricle at the apex of the optic chiasm and is made up of tightly packed neurons of small diameter. It has a dorsomedial (DM) part called the shell/shell and a ventrolateral (VL) part called the nucleus/core

Light acts through retinal ganglion cells containing melanopsin, and subcortical light information is transmitted to the SCN *via* the retino-hypothalamic tract [56].

## Biological rhythms and the cholinergic system in the regulation of locomotor activity

The generation of the rhythmic pattern controlling locomotion is formed by the activity of intrinsically oscillating interneurons in the spinal cord [57]. Locomotion is generated centrally in the spinal cord by circuits referred to as central pattern generators (CPGs). CPGs are triggered by descending commands from the brain (initializing or halting). In addition to the on-demand triggering of these commands to meet the homeostatic needs of the organism, locomotion itself reveals a diurnal pattern directed by a series of pacemakers. The main circadian pacemaker is localized in the hypothalamic SCN [58]. However, some other brain structures have been recently identified as important in locomotor biological rhythm regulation: the subparaventricular zone (SPVZ), intergeniculate leaflet (IGL) [59,60], posterior hypothalamic area (PHA) [60,61], and the tuberomammillary nucleus [61,62]. The SCN is innervated by cholinergic nerves [63], but does not necessarily need to be intrinsically cholinergic [64]. It receives cholinergic projections from the basal forebrain and brain stem tegmentum [65]. The SCN cholinergic fibers and terminals are less dense when compared to other hypothalamic nuclei [63]. PCR has identified all five mAcChRs subtypes in rats [66]. There are species differences in the presence of cholinergic neurons in the SCN in rat, hamster, and mouse [63]. The direct projections [67] originating in the retina directly to the SCN (retinohypothalamic tract), and retinal neurons form axon collaterals to the IgL [68] and SPVZ [59]. In addition to that, SCN receives photic input [67] from the IgL (geniculohypothalamic tract). IgL is a typical structure with a huge spectrum of projections: first, there are connections to the SCN, and also to other structures, like the SPVZ, anterior hypothalamic area, pretectal area, paraventricular thalamus [69], lateral hypothalamus, posterior hypothalamus, anterodorsal thalamic nuclei, centromedial thalamic nuclei, centrolateral thalamic nuclei, anterior paraventricular thalamic nuclei, olfactory tubercle, lateral olfactory tract nuclei, and many others [59]. The SPVZ receives input from the SCN, as does the PHA [70,71]. Vice versa, the SCN receives input from the SPVZ [72]. The SPVZ projects to the thalamus [73]. The paraventricular nucleus of the thalamus receives input from the SCN and also sends efferent projections back to the SCN [74]. The PHA sends neurons to the cortex [61]. Other connections (thalamostriatal, striatocortical, thalamocortical) are well-known and represent textbook knowledge. The cholinergic innervation of the SCN originates in the cholinergic forebrain and brain stem nuclei. The projections from the lower SPVZ to the SCN are cholinergic [75], similar to efferent SCN projections that are responsible for the activation of arousal-promoting cells [76]. As demonstrated earlier [77,78], M_4_ mAcChRs are abundant in the striatum (46 % of all mAcChRs). There was also a relatively high density of M_1_ mAcChRs (37 %). Similarly, a high percentage of M_4_ mAcChRs (43 %) is present in the thalamus. However, the total muscarinic population in the thalamus is low.

It has been shown that there is some rhythmic activity of mAcChRs and enzymes, both synthesizing and degrading, in the cholinergic system. The results of the studies conducted vary widely, although some differences are based on the different species and structures studied. The cholinergic enzymes include choline acetyl-transferase (ChAT) and acetylcholinesterase (AChE). The activity of these enzymes exhibits various circadian irregularities that have been postmortem measured in the human brain [79]. In another investigation of cholinergic enzyme activity and acetylcholine release, it was found that in most brain regions, there is a peak in ACh release and ChAT activity during the active phase of the biorhythm and a peak in AChE activity during the inactive phase [63].

The mAcChR studies report mixed findings. In [79], levels of mAcChRs measured postmortem in the human cerebral cortex were significantly lower during the day than during the night [79]. In a review by [63] the authors reported that in most brain regions, mAcChR levels peak during the inactive phase. In studies or reviews of another research group [80,81], a peak of circadian rhythm in the rat forebrain was observed during the dark phase of the diurnal cycle, i.e., in the active part of the day. In addition, these authors [80] found another peak in the light phase at the brain membranes. Some structures (hippocampus and hypothalamus) in another study [82] revealed circadian rhythmicity in the mAcChRs, while others (cerebral cortex, striatum, or cerebellum) do not. Other brain structures (bulbus olfactorius, parietal cortex, and caudate-putamen) revealed mAcChRs biological rhythms [83] with a peak at subjective night, while no rhythms were identified in the frontal or occipital cortex, nucleus accumbens, hippocampus, thalamus-septum, pons-medulla, or cerebellum. The peak mAcChRs in the inactive period of the rat (at 14:00 h) were determined by saturation studies [84]. In the occipital cortex of Syrian hamsters, circadian rhythms of mAcChRs have been shown, whereas no detectable rhythm was found in the anterior hypothalamus, including the suprachiasmatic nucleus [85].

## Sex differences in biological rhythms

There is evidence of correlations between circadian timing systems and sex differences [86].

In each sex, the same basic neural pathways are present, but they are represented differently (e.g., different numbers of neurons, projections, dendritic spines, and different densities of synapses). Thus, the transmission and processing of pieces of information through sexually dimorphic neural networks is likely to be different in males and females [87]. There is also evidence that sex differences in circadian timing are important for responses to various endogenous and exogenous factors, with implications for understanding behavior and physiology [88]. Differences between females and males in regulation at the level of individual brain cells can arise from many factors, including differences in sex chromosomes, specializations in receptor expression, and ion channels, or as a result of differences in circulating hormones. These cell-based sex differences can, in turn, lead to differences between men and women in brain networks, organs, and behavior. Such effects have substantial implications for applying basic research findings to practical problems and exploring the causes of sex differences in disease incidence [89].

As there are three different components involved in the ability of the SCN to serve as a brain clock (input from the environment *via* retinohypothalamic tract, oscillatory clocks in the SCN, and output pathways to various target areas), all of them can express estrogen receptors, androgen receptors, or both. This allows feedback from circulating hormones to act on each of these components of the circadian timing system. Thus, sex differences in the circadian timing system may arise from the organizing effects of hormones in the pre- and perinatal periods or the activating effects of hormones during puberty and adulthood [86].

It has previously been shown that some aspects of locomotor activity, like the light-dark transition test, running wheel, elevated plus maze (all [90]), and the open field test [91], are affected by sex steroid hormones. The effect on locomotor activity is mediated by estrogen receptor alpha. Also, the differences exist in circadian gene expression [92]. Concerning the mechanisms, female sex steroids (estrogen) have been shown to increase locomotor activity [93] and in the open field [90]. There is a large body of evidence for the influence of sex hormones on the central cholinergic system [93]. For example, the activity of several cholinergic markers, such as ChAT, high-affinity choline uptake, ACh release [94] or AChE [95]. Morphological differences between the males and females have also been found in the size of cholinergic cells [96], differences in the volume of the medial preoptic area and SCN [97], and the number of mAcChR binding sites [98]. At the cellular level, *in situ* hybridization/immunocytochemical studies have shown the co-localization of estrogen receptors and ChAT on cholinergic neurons of the basal forebrain in adult mice and rats [99]. In the case of locomotor activities, most studies predominantly use male rodents, as is the case in M_4_ KO studies. Thus, in addition to genetic background, sex differences in experimental conditions may then contribute to inconsistencies in results regarding the role of muscarinic receptors in locomotion control [100].

## The novel functions of M_4_ mAcChRs

As mentioned above, locomotor activity is influenced by M_4_ mAcChRs. However, a systematic study on the changes in locomotor activity in the diurnal rhythm was not performed. Thus, we have focused on changes in activity and biological rhythm in M_4_ KO animals. To judge if there is an effect on other parameters that reveal biological rhythmicity (i.e., temperature), we have also followed the changes in temperature biological rhythm. The effects were studied separately in males and females.

**Fig. 1:**
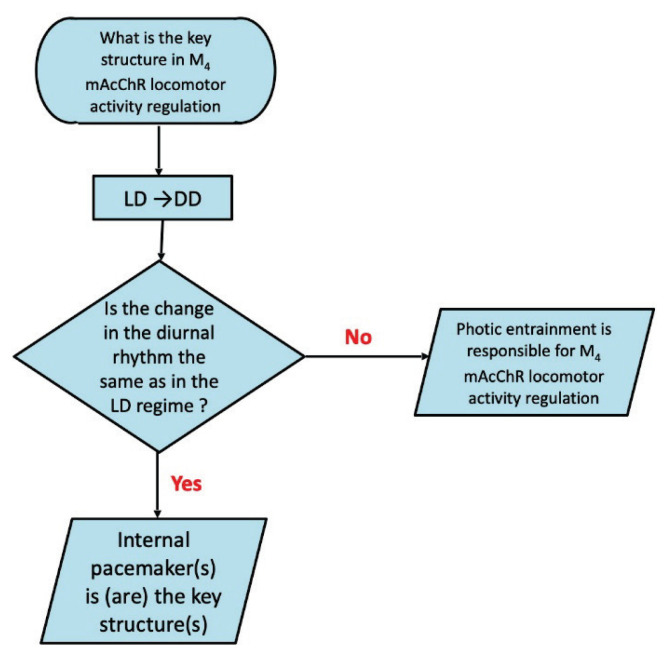
Flowchart (algorithm) for determining if the changes are caused by photic entrainment or are done by internal pacemaker(s). LD: light/dark regime, i.e., 12 h light/12 h dark. DD: dark/dark regime, i.e., constant darkness.

**Fig. 2:**
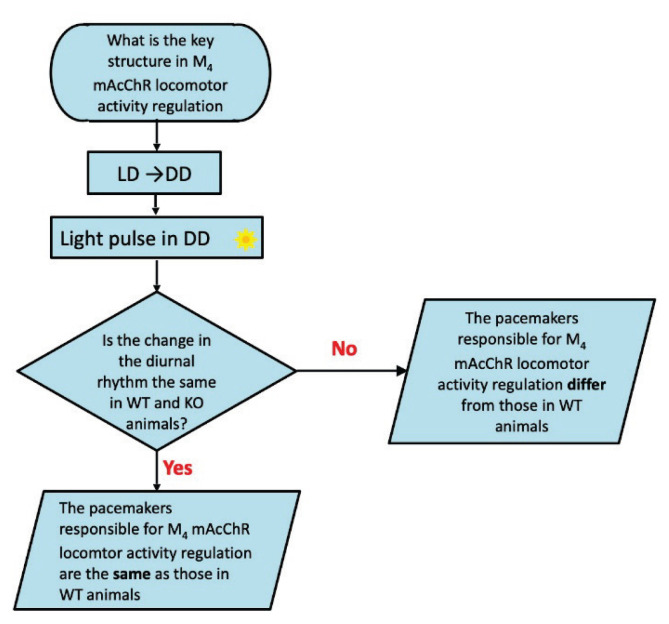
Flowchart (algorithm) for determining if the changes are caused by the same structures in the brain. LD: light/dark regime, i.e., 12 h light/12 h dark. DD: dark/dark regime, i.e., constant darkness. Light pulse in DD: 300 lx for 1 h.

We have not found differences in biological rhythm, either of temperature or locomotor activity [101] in M_4_ KO males. Similarly, the biological rhythm of temperature was unchanged in M_4_ KO females [101]. In detail, motor activity in female M_4_ KO mice did not differ significantly from that of wild-type mice during the light period. However, in the dark phase (the active part of the day in mice), M_4_ KO mice revealed an increase in locomotor activity, which resulted in significant differences in biological rhythm parameters: mesor, nocturnal values (mean), and night-day mean difference were increased, and other parameters were changed in connection with these changes [101]. These data are new and can be explained by the differences in the genetic background, backcrossing and both sexes observation (please compare to [29,31,44,46–48]). Thus, we would like to know if the changes in locomotor activity biological rhythm are caused by pacemaker structures involved in the locomotion regulation or if it is caused by external stimuli, i.e., *via* light/dark regime. Moreover, we would like to know which structures are responsible for these changes [77]. The flow chart is as follows (Figs 1 and 2). Thus, two experiments were performed: in the first (Fig. 1), we placed mice in constant darkness. If the changes in the locomotor activity in M_4_KO mice are the same after switching to constant darkness, then the biological rhythm change in M_4_ KO mice originates in pacemaker structures. In the second experiment (Fig. 2), we use a pulse after the switch into constant darkness to show if the structures involved in the biological rhythm regulation in M_4_ KO mice are the same or not.

**Fig. 3:**
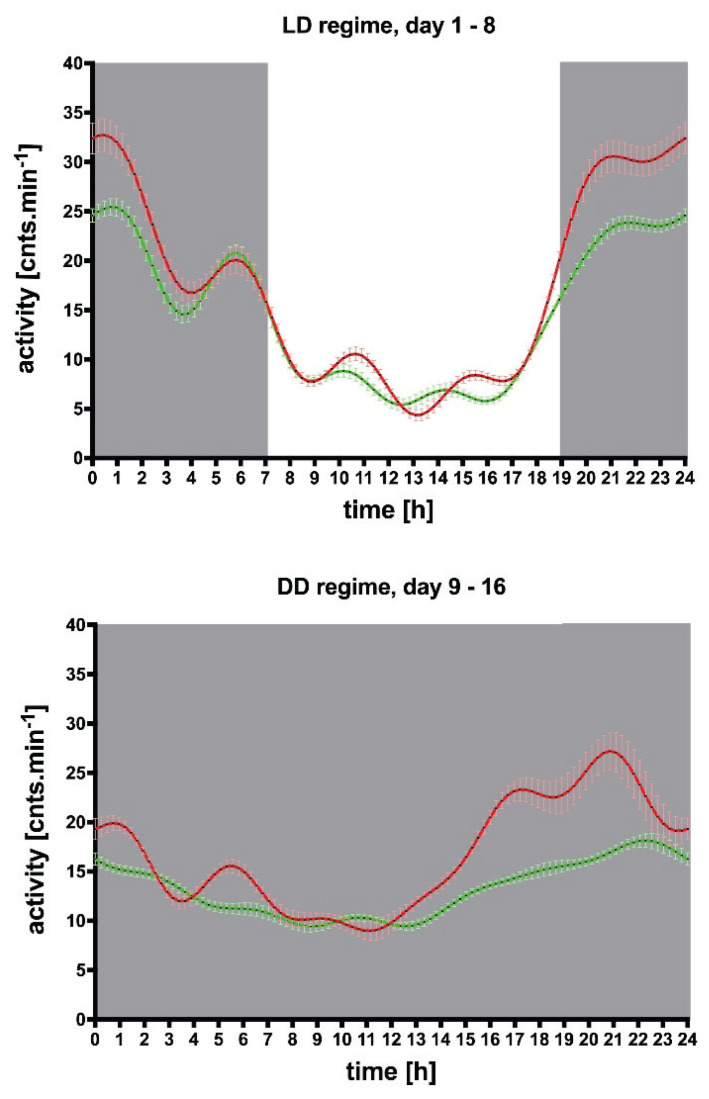
Unchanged biological rhythm after switching to constant darkness. **Top:** The locomotor activity biological rhythm in the LD regime (days 1–8), and in the DD regime (days 9–24), **bottom**. Gray parts represent the dark phase. Abscissa: time (h), ordinate: activity [cnts.min^−1^]. See legend for symbol explanation. From *Brain Structure and Function (2020) 225:1615*–*1629*. © Vladimir Riljak, Katerina Janisova, and Jaromir Myslivecek.

**Fig. 4:**
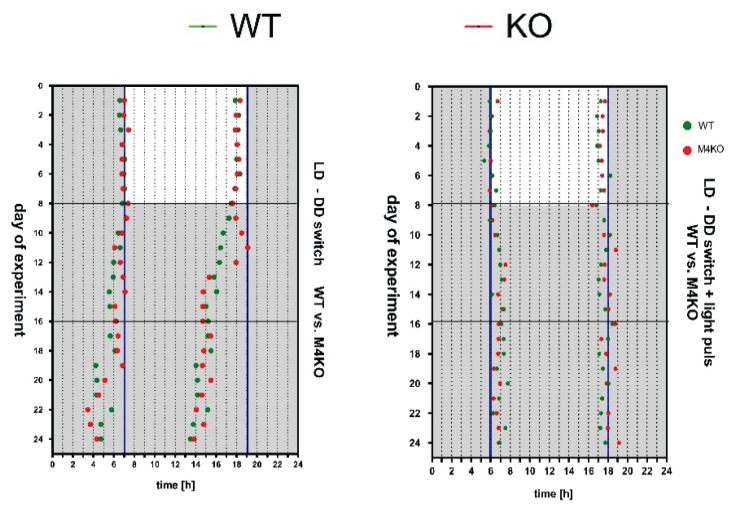
**Left:** Timeshift after the switch to the DD regime expressed as t_MinSlope_ and t_MaxSlope_ in WT and KO animals. Horizontal lines represent the division of time slots (days 1–8, 9–16, 17–24). See legend for symbol explanation. **Right:** time shift after switching to the DD regime with a light pulse (300 lx) applied on day 9, expressed as t_MinSlope_ and t_MaxSlope_ in WT and KO animals. Horizontal lines represent the division of time slots (days 1–8, 9–16,17–24). See legend for symbol explanation. From *Brain Structure and Function (2020) 225:1615*–*1629*. © Vladimir Riljak, Katerina Janisova, and Jaromir Myslivecek.

The increased locomotor activity persisted after the switch to constant darkness, and the biological rhythm parameters were similarly different (Fig. 3) after the switch [77]. Similarly, timeshift after the switch to constant darkness, expressed as t_MinSlope_ and t_Maxslope_ were similar to these parameters in the switch to constant darkness with light pulse (Fig. 4). These results confirm that the M_4_ mAcChRs directed changes in locomotor activity biological rhythm originate in pacemaker structures and are not caused by photic entrainment. Moreover, the structures involved in the M_4_ mAcChRs directed changes in locomotor activity and biological rhythm are probably the same in WT and KO animals.

**Fig. 5:**
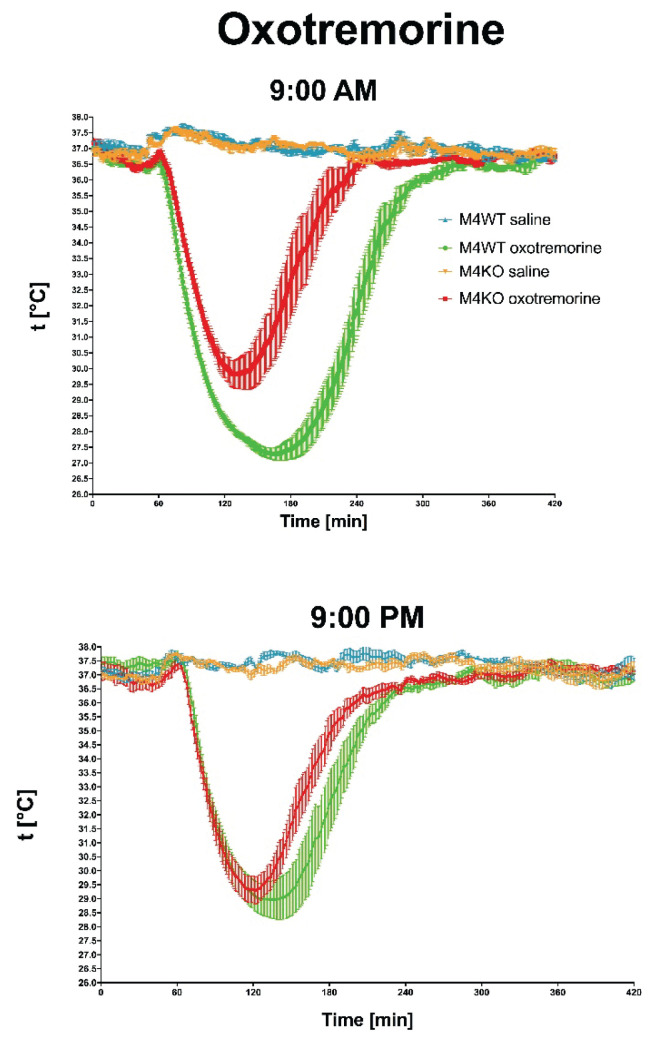
The effects of oxotremorine/saline on temperature 360 min after oxotremorine application. The temperature was also monitored 60 min before drug application. Ordinate: t [°C], temperature. Abscise: time [min]. Top: the effects at 9:00 AM (oxotremorine/saline application); bottom: the effects at 9:00 PM (oxotremorine/saline application). No changes in temperature were recorded after saline application. From *Front Pharmacol (2019) 10:237. doi:10.3389/fphar.2019.00237*. © Paulina Valuskova, Vladimir Riljak, Sandor T. Forczek, Vladimir Farar, and Jaromir Myslivecek.

**Fig. 6:**
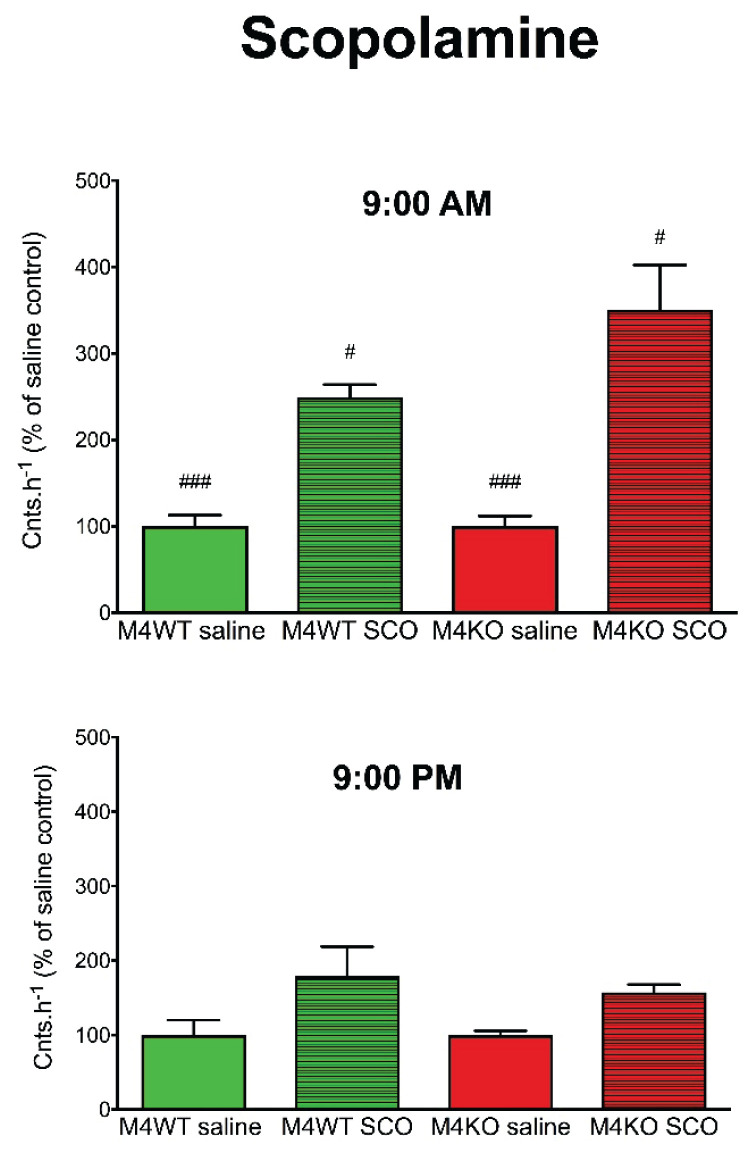
The effects of scopolamine (SCO) on locomotor activity in WT (M4 mAcChR WT) and KO (M4 mAcChR KO) mice at 9:00 AM (top) and 9:00 PM (bottom). Control mice were treated with a physiological solution (saline). Ordinate: counts per hour expressed as % of activity in saline-injected mice. ^#^ p<0.05, ^###^ p<0.001, difference from PM. From *Front Pharmacol (2019) 10:237. doi:10.3389/fphar.2019.00237*. © Paulina Valuskova, Vladimir Riljak, Sandor T. Forczek, Vladimir Farar, and Jaromir Myslivecek.

**Fig. 7:**
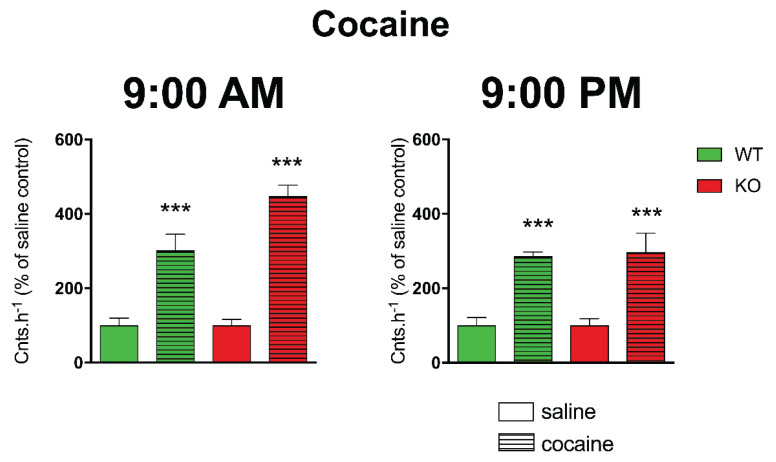
The effects of cocaine on locomotor activity in WT and KO mice at 9:00 AM (left) and 9:00 PM (right). Control mice were treated with a physiological solution (saline), see legend in the figure. Ordinate: counts per hour expressed as % of activity in saline-injected mice. *** p<0.001, difference from saline-injected mice. From *Front Pharmacol (2019) 10:237. doi:10.3389/fphar.2019.00237*. © Paulina Valuskova, Vladimir Riljak, Sandor T. Forczek, Vladimir Farar, and Jaromir Myslivecek.

Another aspect that is in close relation to biological rhythm, to locomotor activity, respectively, is the characteristics of animals as diurnal or nocturnal. As is widely known, mice are nocturnal animals. Thus, they are active at night (dark period) and they sleep in the day (light period). However, many experiments are performed during the day, i.e., when mice normally sleep. Thus, we would like to know if there will be any difference when we apply muscarinic drugs (agonist: oxotremorine, antagonist: scopolamine), and we will follow the effects in the morning (at 9:00 AM) and in the evening (at 9:00 PM). For comparison, we have also applied a drug able to increase locomotor activity but affecting other (dopaminergic) mechanisms, i.e., cocaine. While both oxotremorine and scopolamine differed in their effects in the morning vs. evening [100], cocaine had similar effects when administered at 9:00 AM or 9:00 PM (Figs 5, 6, 7).

**Fig. 8:**
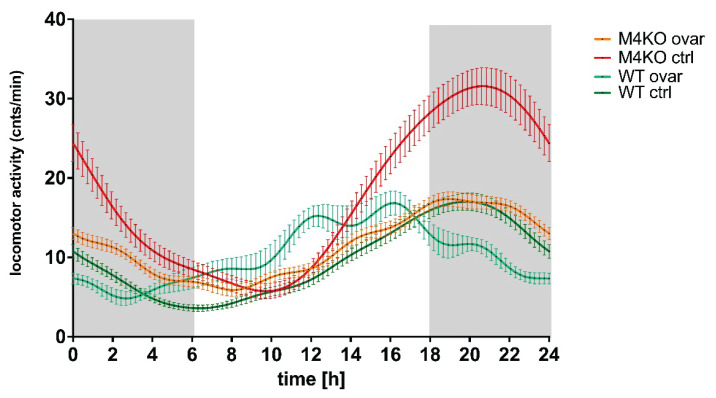
The changes in locomotor activity in ovariectomized mice. While in WT animals the ovariectomy did not cause a change in biological rhythm, in M_4_ KO, there was a decrease in locomotor activity to control (WT) values, giving evidence that M_4_ mAcChRs are essential in locomotor activity biological rhythm regulation. Abscissa: time [h], ordinate: activity [cnts.min^−1^]. Gray parts represent the dark phase. See legend for symbol explanation.

As mentioned above, the sex hormones can affect the biological rhythms. Thus, we have used ovariectomy to compare the effects in M_4_ KO and WT animals. As expected, ovariectomy in M_4_ KO decreased the typical locomotor activity increase in the dark (active) period in females, while in WT, there was no effect (Fig. 8). These data show that sex hormones can affect the locomotor activity biological rhythm and could explain why there is no change in locomotor activity biological rhythm in males. According to our knowledge, the sex differences were not studied yet in the M_4_ mAcChR directed biological rhythm of locomotion.

To search specific structures involved in M_4_ mAcChR, we have studied the changes in M_4_ mAcChR density in specific brain areas (SCN, SPVZ, PHA, IgL, thalamus, striatum, and motor cortex). We also followed changes in M_1_ mAcChR density in the striatum and motor cortex and changes in the activity of AChE and BuChE [102]. We have found that initially, the ultradian rhythm in SCN is changed to circadian in SPVZ, PHA, and IgL. In these structures, there is a time shift between the rhythm in WT and M_4_ KO animals. Similar rhythms exist in the thalamus and motor cortex. The key structure for M_4_ mAcChR locomotor activity regulation is the striatum, where it is possible to find the correlation between the biological rhythm of receptor densities, both in WT and KO animals, and locomotor activity.

## The clinical implications of novel M_4_ muscarinic functions

Cholinergic receptors have been implicated in schizophrenia, Alzheimer’s disease, Parkinson’s disease, and Huntington’s disease. The striatal cholinergic neurons are tightly connected with the dopaminergic system, and the balance between these systems is essential for appropriate locomotor response [40]. The striatal medium spiny neurons subpopulation expresses D_1_ dopamine receptors and is affected by the M_4_ mAcChR [103]. The locomotor response is also mediated by M_1_ mAcChR. The data on the simple effects of M_1_/M_4_ mAcChR on locomotor activity were reported regularly. As mentioned before, M_4_ mAcChRs have been shown initially to influence locomotor activity. On the other hand, the subsequent studies, in which backcrossing was carefully performed on C57Bl/6J background, showed no basal M_4_ effects on motor activity. It is therefore possible to conclude that M_4_ mAcChR affects locomotion. However, the role of M_4_ mAcChR in the sex-specific biological rhythm is new. The locomotor response is also influenced by nicotinic acetylcholine receptors [104].

Interestingly, vesicular acetylcholine transporter (VAChT) mutant mice with increased ACh in the brain present preserved neuromuscular function, but altered brain cholinergic function, and are hyperactive. Genetic removal of the resistance cassette rescues VAChT expression and the hyperactivity phenotype [105]. Elimination of VAChT had only marginal consequences in striatum-related tasks and did not affect spontaneous locomotion [106]. This group also found that up to 50 % a decrease in VAChT expression in the brain does not change locomotor activity in mice, a more pronounced decrease in VAChT expression causes hyperactivity in a new environment. On the other hand, we have not found significant differences in locomotion (hyperactivity) in mice with a complete lack of CNS AChE (PRiMA KO mice) and thus almost 300 times increased Ach in the striatum [6].

As mentioned above, the locomotor response is also influenced by M_1_ mAcChR. In this paragraph, we will describe the role of other muscarinic subtypes, i.e., M_1_, M_2_, M_3_, and M_5_ mAcChR, in locomotion. In that context, it has been shown that M_1_ mAcChR play an important role in the regulation of locomotor activity but appear to be less critical for cognitive processes, as generally assumed [107]. The lack of M_1_ mAcChR or M_2_ mAcChR had no significant effect on the magnitude of striatal dopamine release. This response was abolished in M_4_ KO mice, significantly increased in M_3_ KO mice, and significantly reduced (but not abolished) in M_5_ KO mice [108]. It has also been suggested that the dopamine release-stimulating M_4_ mAcChR is probably located on neuronal cell bodies, but that the release-facilitating M_5_ mAcChR and the release-inhibiting M_3_ mAcChR are likely to be located on nerve terminals. M_3_ and M_4_ mAcChR mediate their dopamine release-modulatory effects *via* facilitation or inhibition, respectively, of striatal GABA release [108]. M_4_ mAChRs are coexpressed with D_1_ dopamine receptors in a specific subset of striatal projection neurons. Mutant mice that lack M_4_ mAChRs in D_1_ dopamine receptor-expressing cells only had enhanced locomotor activity and increased behavioral sensitization following treatment with psychostimulants. These behavioral changes were accompanied by a lack of muscarinic inhibition of D_1_ dopamine receptor-mediated cAMP stimulation in the striatum and an increase in dopamine efflux in the nucleus accumbens [109]. Another experimental design targeted to medium spiny neurons bearing M_4_ mAChRs and D_1_ dopamine receptors used DAT knockout mice (with increased dopamine levels) [103]. *In vivo* experiments with AChE inhibitors, as well as with the positive allosteric modulators of M_4_ mAChR, showed that these effects could reverse the increased locomotor activity of DAT knockout mice. The authors conclude that M_4_ mAChR could be a novel therapeutic target to treat hyperactivity disorders [103], which is also in agreement with our results showing the hyperactivity in the dark phase in females. Similarly, the pharmacological influence of M_4_ mAChR can affect NMDA receptor blockade-induced hyperlocomotion. In detail, selective activation of M_4_ mAChR by VU0467154 can reverse NMDA receptor antagonist MK-801-induced hyperlocomotion [30].

As an example, two diseases are connected with prominent changes in locomotion: Parkinson’s disease and ADHD (Attention-deficit/Hyperactivity disorder). Both are affected by M_4_ mAChR. mAChR antagonists can reduce motor symptoms when dopamine release is diminished (e.g., Parkinson’s disease). M_4_ mAChR plays a central role in regulating dopamine signaling and release in the basal ganglia (see above). This raised the possibility that sources of ACh outside of the striatum can regulate motor activity [110]. The novel, time- and sex-dependent functions of M_4_ mAChR could explain differences in locomotion that had not been clear yet. In that context, long-term decreases in the densities of the muscarinic receptor subtypes M_1_, M_2_, and M_3_ (mAchRs), and the nicotinic receptor subtype α_4_β_2_ in the striatum of the hemiparkinsonian rat model [111] can complete the picture of the disease and are in congruence with previously discussed results. Similarly, it is also in agreement with our results on biological rhythm in locomotor behavior.

ADHD is of higher interest to M_4_ mAcChR mAcChR novel functions as there was clear hyperactivity limited to the active period and to female sex. Interestingly, there is an animal model of ADHD that is connected with social isolation. In detail, social isolation Reveals an epigenetic animal model of ADHD in which central cholinergic systems play a role [112]. These experiments have shown acetylcholinesterase inhibitors donepezil and tacrine, as well as the positive allosteric modulators of M_4_ mAcChR, to be sufficient to reverse the increased locomotor activity of DAT knockout mice. This is in good agreement with our results on the M_4_ mAcChR novel function and suggests that M_4_ mAcChR could be a novel therapeutic target to treat hyperactivity disorders [103]. Another report has shown a complete lack of effect of M_1_ mAcChR deletion on measures of attentional function per se, but there were abnormalities on a variety of response measures: M_1_ KO mice displayed fewer omissions, more premature responses, and increased perseverative responding. These data suggest that M_1_ KO mice display abnormal responding in the face of relatively preserved attention, learning, and perception [113]. Similarly, these results also support our findings on the minor role of M_1_ mAcChR in the biological rhythm locomotor regulation. The study on M_4_ KO mice suggested that Dopaminergic dysfunction is an important pathogenetic factor for Parkinson’s disease and ADHD. Loss of M_4_ mAcChR control causes a state of dopaminergic hyperexcitability. These authors also propose that M_4_ mAcChR agonists could be a strategy for the treatment of pathologies associated with hyperdopaminergia [114].

It has also been shown that ADHD reveals cyclic appearance with respect to the severity of symptoms [115,116]. This brings another aspect of novel M_4_ mAcChR functions as a function of time.

## Conclusions

Changes in locomotor activity and biological rhythm caused by M_4_ mAcChR [101] involve multiple brain structures. These potential brain regions have been identified [77], and differences in the effect of substances acting on muscarinic receptors when administered in the morning and evening have been found in M_4_KO animals and WT [100].

**Fig. 9:**
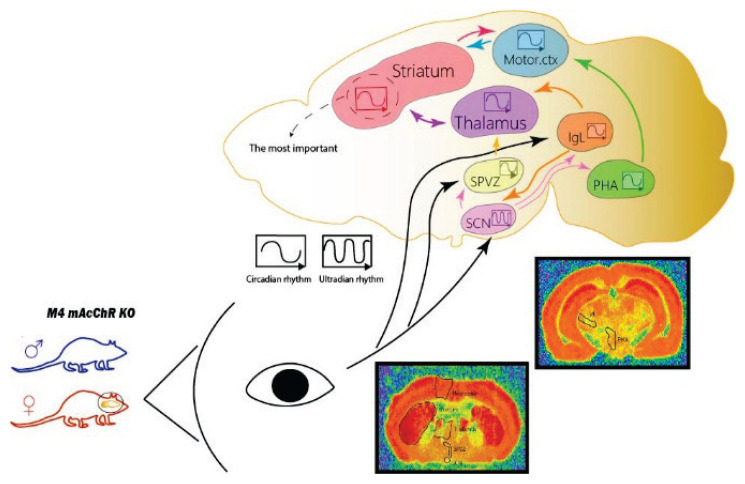
Schematic diagram depicting the role of M_4_ mAcChRs in the regulation of locomotor activity and biological rhythm. While in males (blue mouse) there is no change in locomotor activity in M_4_ KO animals, in females (dark pink mouse) there is an increase in active period locomotor activity. The biological rhythm of mAcChRs responsible for these changes is shown in the right part of the scheme. Symbols for ultradian/circadian rhythm are shown at the in the centre of the Figure. Photic afferenttation causes an ultradian rhythm in SCN, which is transposed to circadian in the subparaventricular zone (SPVZ), posterior hypothalamic area (PHA), and intergeniculate leaflet (IgL) with a time shift. Then, the thalamus and motor cortex reveal a circadian rhythm with the same period, and the striatum (shown by the red line) is the key structure for biological activity rhythm.

Potential mechanisms of changes in locomotor biological rhythms were sought using constant darkness to distinguish light sensitivity from actual circadian effects [77]. We showed that although the output of the core clock is altered by deletion of the M_4_ mAcChR, the structures involved in the regulation of biological rhythm in WT and KO animals are likely to be the same (striatum, thalamus, and intergeniculate leaflet). It has also been found that M_1_ mAcChRs in the striatum are involved in the regulation of locomotor biological rhythm [77]. The probable interplay between the brain structures, as it can be judged from the changes in receptor densities, is shown in Figure 9. We conclude that the key structure in M_4_ mAcChR-directed changes in locomotor activity biological rhythm is the striatum.

## Abbreviations

Ach: acetylcholine
AChE: acetylcholinesterase
ADHD: Attention-deficit/Hyperactivity disorder
BuChE: butyryl-cholineesterase
cAMP: cyclic adenosine mono-phosphate
CNS: central nervous system
D1: dopamine receptors type 1
D2: dopamine receptors type 2
D3: dopamine receptors type 3
D4: dopamine receptors type 4
D5: dopamine receptors type 5
DAT: dopamine transporter
GPCRs: G-protein coupled receptors
ChAT: choline acetyltransferase
IgL: intergeniculate leaflet
mAcChRs: muscarinic acetylcholine receptors (M_1_, M_2_, M_3_, M_4_, and M_5_)
M_4_ KO: mouse with a deleted muscarinic receptor type 4 gene
PHA: posterior hypothalamic area
SCN: suprachiasmatic nuclei
SPVZ: subparaventricular zone
WT: Wild Type mice
